# A Metagenomic Framework for the Study of Airborne Microbial Communities

**DOI:** 10.1371/journal.pone.0081862

**Published:** 2013-12-11

**Authors:** Shibu Yooseph, Cynthia Andrews-Pfannkoch, Aaron Tenney, Jeff McQuaid, Shannon Williamson, Mathangi Thiagarajan, Daniel Brami, Lisa Zeigler-Allen, Jeff Hoffman, Johannes B. Goll, Douglas Fadrosh, John Glass, Mark D. Adams, Robert Friedman, J. Craig Venter

**Affiliations:** 1 Informatics, J. Craig Venter Institute, San Diego, California, United States of America; 2 Synthetic Biology and Bioenergy, J. Craig Venter Institute, Rockville, Maryland, United States of America; 3 Microbial and Environmental Genomics, J. Craig Venter Institute, San Diego, California, United States of America; 4 Informatics, J. Craig Venter Institute, Rockville, Maryland, United States of America; Wilfrid Laurier University, Canada

## Abstract

Understanding the microbial content of the air has important scientific, health, and economic implications. While studies have primarily characterized the taxonomic content of air samples by sequencing the 16S or 18S ribosomal RNA gene, direct analysis of the genomic content of airborne microorganisms has not been possible due to the extremely low density of biological material in airborne environments. We developed sampling and amplification methods to enable adequate DNA recovery to allow metagenomic profiling of air samples collected from indoor and outdoor environments. Air samples were collected from a large urban building, a medical center, a house, and a pier. Analyses of metagenomic data generated from these samples reveal airborne communities with a high degree of diversity and different genera abundance profiles. The identities of many of the taxonomic groups and protein families also allows for the identification of the likely sources of the sampled airborne bacteria.

## Introduction

Air is an important transport medium for microbes, allowing them to overcome geographic barriers and disperse over long distances [1]–[3]. While airborne microbes were initially studied in the context of disease transmission [3], the role of bacteria in atmospheric processes like cloud formation is becoming evident [4], [5]. Furthermore, it is also evident that air can serve as a habitat for certain microbes that are able to metabolize compounds found in the atmosphere for growth and reproduction [6]–[8]. While most of our understanding of aerosol microbiology has been obtained from the study of bacteria and fungi that can be cultivated, it is also known that cultivable microbes make up only a small fraction of the total airborne microbes [1]. Overall, airborne microbial community composition and dynamics remain poorly characterised, particularly in the indoor environment where humans in industrialized countries spend nearly 90% of their time [9]. The development of new approaches for comprehensive characterization of airborne microbial communities thus has the potential to impact various disciplines, including studies of microbial diversity and biogeography, public health, building design, and understanding microbial role in biogeochemical cycling and climate processes.

Airborne environments, however, have extremely low density of biological material. Concentrations of atmospheric bacteria over land are estimated to be 10^4^ cells/m^3^ (with concentrations over sea lower by a factor of 100–1000) [1], [10]. In contrast, the number of bacteria in surface marine environments has been estimated to be 10^9 ^cells/m^3^ [11], in soil 10^10^–10^11^ per gram of soil [12], and in the human distal gut 10^13^–10^14^ per human [13]. Fungal concentrations in outdoor environments have been reported to be in the 10^3^–10^4^ spores/m^3^ range [14], [15]. Primarily because thousands of liters have to be collected for a representative sample, the majority of non-culture based studies conducted on airborne communities to date have relied on amplification and sequencing of the 16S (for bacteria) or 18S (for fungi) ribosomal RNA gene to characterize their taxonomic composition [16]–[25].

In contrast to targeted sequencing of taxonomic markers, the metagenomic paradigm based on whole genome shotgun (WGS) sequencing of environmental DNA samples, allows for examination and analysis of both taxonomic composition and metabolic potential of the sampled microbial communities [26]. This paradigm has been used to study microbial composition and diversity in many environments [13], [27]–[29].

Here we present a metagenomic framework for studying airborne microbial communities, including protocols for sample collection, and for isolation and amplication of DNA from these samples. A WGS study of airborne microbes requires millions of liters of air be sampled to obtain enough material for sequencing; this requires long sampling times, an efficient means for capture of microbial constituents, and processes to limit post-capture growth which would skew the representation of sampled microbes. We applied this framework to study the composition of indoor and outdoor air samples collected from locations in New York City (NYC), New York and in San Diego (SD), California. Since DNA yields were inadequate for sequencing, the DNA isolated from the samples was amplified to generate sufficient amounts for library construction. Sequence data generated from the libraries were used to study the taxonomic composition of the sampled microbial communities and the functional composition of their bacterial constituents.

## Materials and Methods

### Samplers and Sample collection

Indoor and outdoor air environments were sampled in NYC and SD. Permissions were obtained to collect air samples at each of the locations. Sampling was carried out using SpinCon®PAS 450-10A Wet Cyclone Portable Air Samplers (Sceptor Industries, Kansas City, MO), modified as described below. The samplers were operated at a flow rate of 450 L/min. The sampling buffer for the SpinCon air samplers was comprised of 1X PBS pH 7.0 supplemented with 0.25 mg/ml tetracycline, 0.25 mg/ml gentamycin and 0.125 mg/ml chloramphenicol made up in one liter of sterile water for injection (Baxter, Deerfield, IL). Evaporative loss of the water inside the sampler was off-set with sterile water for irrigation (Baxter, Deerfield, IL), supplied from a 10 L bag. Approximately 10 mls of sample buffer was maintained in the sampling chamber throughout the sampling cycle. The sample cycle time was two hours. After each two hour collection cycle, the sampler was programmed to turn off and drain the sample into a 250 ml corning bottle containing sodium azide (0.02% final concentration after the 12 hour sampling cycle) placed in a modified 4°C mini-refrigerator. The sampler would then refill the sample chamber with sample buffer and restart the collection cycle. Every 12 hours the aggregate sample, comprised of six discrete samples, was removed from the 4°C refrigerator, molecular biology grade glycerol was added to a final concentration of 10% and the sample was stored at −20°C for transport back to the lab and subsequent storage at −80°C. The sampling apparatus and protocol are depicted in Figure S1. Every 24 hours the samplers were subjected to a clean in place (CIP) protocol described below.

### Modification of the SpinCon®PAS 450-10A Portable Air Samplers

All manufacturer supplied tubing and fittings were replaced with antimicrobial fittings and tubing (Cole Parmer, Vernon Hills, IL) where appropriate or completely removed from the sampler. Anti-microbial tubing and fittings were used to feed sample buffer and the make-up water to the sampling chamber.

### CIP Protocol

The SpinCons were disassembled and cleaned as follows. The leads from the makeup water and the sample buffer were closed via a stopcock, capped and removed from the unit. The units were turned off and unplugged. The air inlet and outlet pipes were removed, one end was capped with aluminum foil, and the interior of the pipes were sprayed with 70% isopropanol. The other end was capped and the pipes were set aside until reassembly. The collection chamber and blower unit were removed from the box. All gaskets were removed washed with Liquinox, and rinsed with DI water and air dried. The collector/concentrator was cleaned thoroughly with Liquinox solution, rinsed well in DI water, and sprayed with 70% isopropanol, wrapped in foil and set aside. The peristaltic pump head was removed from the bottom of the sampler and the tubing was replaced with a new piece (30 cm) of autoclaved Pharmed (Cole Parmer, Vernon Hills, IL) tubing (1/8″ OD) with foil wrapped ends. The pump head was then remounted on pump axle. The sampler was reassembled and rinsed as follows: Two manual collections and two rinses were done with 10% Chlorox. The second Chlorox rinse was allowed to remain in the collector/concentrator chamber for 10 minutes before being drained. Two manual collection and two rinses were done with 0.5% sodium thiosulphate. The second sodium thiosulphate rinse was allowed to remain in the sample chamber for 10 minutes before being drained. Two manual collection and two rinses were done with 70% isopropanol. The second isopropanol rinse was allowed to remain in the collector/concentrator chamber for 10 minutes before being drained. The sample buffer was reattached to the unit and one manual collection and one rinse was done. The makeup water was reattached to the unit and the sampling cycle was restarted.

### Material Handling and Controls Used During Sample Preparation

Large volumes of buffers and reagents were UV treated by placing them in a biosafety cabinet with the UV lamp on for 2 hours. When appropriate, caps were removed to allow maximum exposure to the UV light. Liquid volumes less than 500 µl were UV treated in a Stratalinker 1800 for 10 minutes. Tubes used during the extraction of the filters were treated with Sigmacote, rinsed with UV treated water and air dried in a biosafety cabinet. The tubes were then UV treated in a Stratalinker. Low binding, UV treated microfuge tubes (Ambion, Grand Island NY) were used during the purification and concentration of the DNA. All gel rigs were treated with DNAaway (Molecular BioProducts, San Diego, CA) and rinsed with UV treated water. Dedicated pipets and low binding pipet tips were used for all of the protocols. All of the enzymes used in the purification and amplification of the DNA were treated with 0.125-0.5 units of nuclease S7 (Roche Applied Science, Indianapolis, IN) for one hour at 30°C in the presence of 5 mM CaCl_2_. Treated enzymes were stored at −20°C in UV treated tubes. Prior to addition of the enzyme to a reaction, the enzyme was incubated at room temperature for 10 minutes in an appropriate reaction buffer supplemented with EGTA to inactivate the nuclease. DNA and/or random hexamers was then added to the reaction. Positive controls during the MDA and linker amplification steps consisted of purified genomic DNA, while no template negative controls were used to assess spurious amplification and DNA contamination. No template control lanes in verification gels were post-stained with SYBR Gold and visualized using a Typhoon (GE Healthcare Life Sciences, Piscataway, NJ), and the gels were overexposed to show no detectable background amplification. Controls were also employed during the amplification of the 16S V3–V5 variable region.

### Fractionation of the Particulates

Individual 12 hour indoor or outdoor samples were thawed overnight at 4°C. The samples were then transferred to a biosafety cabinet and the outside of the sample bottle was sprayed with 70% isopropanol and allowed to air dry. Samples were transferred to 50 mL conical tubes. De-aggregation of the particulates was accomplished by adding Tween-20 to a final concentration of 0.003%, incubation with inversion on a LabQuake Rotisserie at 37°C for 30 minutes, sonication at room temperature for 10 minutes on the highest setting in a bath sonicator (Aquasonic Model 50D, VWR) and a final incubuation with inversion at 37°C for 30 minutes. Samples were then filtered through an autoclaved 25 mm Versapor 3.0 µm filter (Pall Life Sciences, Ann Arbor, MI) followed by filtration through an autoclaved 25 mm Supor 0.1 µm filter (Pall Life Sciences, Ann Arbor, MI). The filters were folded in half sample side in and transferred to vacuum seal bags and stored at −80°C. The 0.1 µm filtrate was stored in 50 ml conical tubes at −80°C and not used for the studies presented here.

Our pilot sampling efforts conducted in the spring and summer months of 2005–2006 used a DNA isolation method based on that used by the Global Ocean Sampling (GOS) project [27], [30]. DNA isolated from this sampling attempt was not refractory to enzymatic treatment. However, initial attempts at DNA isolation from the NYC November 2007 samples using the GOS DNA isolation method yielded DNA that was refractory to enzymatic manipulation including amplification by PCR or MDA. This was likely a result of the presence of magnetic particulates, possibly iron, in the samples. It has been reported [31] that auto-oxidation of iron in the presence of Tris buffers is greatly enhanced, leading to free radical formation which can potentially damage DNA. It has also been noted [32] that heavy metals present in both coarse and fine particulate matter in urban air samples caused DNA damage. In light of these observations, the DNA extraction protocol was changed to the method described below, which removed the Tris and the EDTA from the extraction buffer. Out of an abundance of caution, the extracted DNA was placed on a magnet to remove particulates and drop dialyzed. The DNA was then treated with PreCR (New England BioLabs, Beverly, MA) to repair any DNA damage. In order to maximize DNA recovery, the filters were dissoved and the DNA was pooled with the DNA from the initial filter extraction.

### DNA Isolation

Individual filters were removed from the −80°C and allowed to thaw at room temperature inside a biosafety cabinet. Thawed filters were aseptically cut into small pieces and placed in individual silanized tubes. Powersoil bead reagent (MoBio Laboratories, Inc. Carlsbad, CA) was added to just cover the filter pieces (300–400 µl). Sixty µl of Powersoil C1 and Proteinase K (200 µg/ml final concentration) were added and the sample was incubated at room temperature for one hour. The sample was gently mixed every twenty minutes. An aliquot of Proteinase K equal to the first aliquot of Proteinase K was added (a final concentration of 400 µg/ml) and the sample was subjected to three freeze/thaw cycles in dry ice/ethanol and a 55°C water bath. Samples were incubated at 55°C for two hours with rotation at 175 rpm. The sample was recovered from the filter pieces and transferred to microfuge tubes. Recovery of nucleic acids was accomplished by adding sodium chloride to a final concentration of 0.25 M, 15 mg of glycoblue (Ambion Grand Island, NY) and an equal sample volume of isopropanol. The precipitation was carried out overnight at room temperature. The DNA was recovered from the isopropanol by spinning at >20 K rpm at room temperature for one hour. Pellets were washed twice with 70% ethanol and resuspended in TE. The samples were phenol extracted twice followed by extraction with phenol:chloroform:isoamyl alcohol. The nucleic acids were isopropanol precipitated overnight at room temperature in the presence of sodium acetate (0.3 M final concentration). The DNA was recovered from the isopropanol by spinning at >20 K rpm at room temperature for one hour. Pellets were washed with 70% ethanol and resuspended in TE. Pellets were pooled with the DNA recovered from the filter dissolution (see below).

### Filter Dissolution

Extracted filters were resuspended in a volume of Powersoil bead reagent (MoBio Laboratories, Inc. Carlsbad, CA) to just cover the filters and 0.5 volume of phenol was added. For the 0.1 µm filters 1.5 volumes of chloroform was added, for the 3.0 µm filter, 2 volumes of chloroform was added. The filters were then incubated with inversion at 65°C for 10 minutes. The phases were separated by centrifugation and the aqueous phase was recovered. The aqueous phase was extracted twice with phenol:chloroform:isoamyl alcohol (25∶24∶1 v/v). DNA was precipitated as described above and pellets were combined with the initial DNA extraction.

### Removal of Magnetic Particulates (NYC samples only)

The combined sample (from the initial extraction and the filter dissolution) was placed on a magnet (Life Technologies, Grand Island, NY) for 20 minutes at room temperature. The solution containing the DNA was then carefully removed and the tube was removed from the magnet. The magnetic particulates were washed with 50 µl of TE and placed on the magnet for an additional 20 minutes. This wash was pooled with the initial solution and drop dialyzed.

### Drop Dialysis (NYC samples only)

Approximately 100 mls of MilliQ water (enough to cover the bottom) was placed in the bottom of an oblong Pyrex dish and heated on high power in a microwave for 1 minute. The dish was covered and allowed to cool slightly. A small petrie dish was placed in the pyrex dish and 30 mls of 1/5X TE was added to the petrie dish. A 0.025 µm Millipore (Millipore, Billerica, MA) disc was floated on top of the 1/5X TE and the DNA sample was carefully added to the disc. Dialysis was carried out at RT for 45 minutes. Samples were recovered from the disc and the spot where the sample was on the disc was washed with an additional 50 µl of 1/5 X TE. The sample was then placed in a Speed-Vac and evaporated until approximately 10 µl remained in the tube. Samples were pooled according to filter size and location. Yield was estimated on a 1 mm thick vertical 1% agarose gel post-stained with Sybr gold (Life Technologies, Grand Island, NY) and visualized on a Typhoon.

### Repair of Damaged DNA

Gel purified DNA was treated with Pre-CR (New England Biolabs, Beverly, MA) as follows: a mastermix comprised of 5 µl of 5X Thermopol buffer supplemented with 50 mM EGTA and 2.5 µl of treated PreCR enzyme mix was incubated at room temperature for 10 minutes. 0.5 µl of 100X NAD+, 0.5 µl of 10 mM dNTP's, and 12.5 µl of water was added to the mastermix. 5 µl of the mastermix was added to 5 µl of DNA. The reaction was incubated at 37°C for 20 minutes followed by overnight incubation at 4°C. The treated DNA was phenol extracted, followed by a phenol:chloroform:isoamyl alcohol (25∶24∶1 v/v) extraction, followed by ethanol precipitation. For post MDA and debranching this reaction was scaled by a factor of 5 and the reaction was carried out at 37°C for one hour before extraction and precipitation.

### phi29 Amplification of Samples (NYC samples only)

DNA was amplified using the Illustra Genomophi (GE Healthcare Life Sciences, Piscataway, NJ) enzyme cocktail following a modified version of a previously described protocol [33]. The final reaction buffer (G-Buffer) was comprised of 37 mM Tris-Cl pH 7.5, 10 mM MgCl_2_, 5 mM (NH_4_)_2_SO_4_, 50 mM KCl, 1 mM each dNTP's, 10 mM EGTA, 4 mM DTT, 0.04 mM random hexamers (IDT) 0.2% Tween 20 and 100–1000 pg of input template. Ten reactions were set up for each sample to minimize any bias that might result from the amplification. The positive control consisted of 100 pg of DNA isolated from a 0.1 µm marine metagenomic filter [30]. The amplification protocol was performed in a thermocycler and was modified as follows: samples were heated to 70°C for 5 minutes and then quickly cooled to 4°C. Samples were removed to ice and opened in a biosafety cabinet where the enzyme master mix was added. The samples were briefly mixed and returned to the thermocycler. It has been reported that high G + C% regions of DNA are preferentially amplified during amplification with phi29 polymerase [34]. One possible explanation for this is that the 30°C annealing and amplification temperature that is used favors G + C hexamer annealing. To minimize this effect, samples were incubated at 4°C for 10 minutes. A temperature ramp of 0.1°C/sec was applied to the samples until 10°C was reached; incubation at 10°C was for 10 minutes. This ramp and incubation was repeated at 15°C, 20°C, and 25°C with a final 0.1 C/sec ramp to 30°C. One hundred picograms of DNA isolated from a 0.1 µm marine metagenomic filter served as the positive contol; a no template control was also included. Samples were then incubated at 30°C for 1.5 hours, followed by a 10 minute 65°C incubation. The yield from individual reactions were assessed by agarose gel electrophoresis. Approximatedly 1/10^th^ of each reaction containing template or the entire no template control was visualized on a 0.8% e-gel. DNA from lanes containing input template were clearly visible with ethidium bromide staining. To assess the no template control lanes the gel was post-stained with SYBR Gold and visualized with a Typhoon. Over exposure of the gel showed no detectable background amplification. Amplified DNA was pooled, phenol:chloroform:isoamyl alcohol (25∶24∶1 v/v) extracted and ethanol precipitated overnight at room temperature in the presence of 0.3 M sodium acetate and 15 mg glycoblue. Pooled samples were washed with 70% ethanol and resuspended in TE. The yield from the MDA was determined by reading the A_260_ using a Nanodrop ND-1000 spectrophotometer.

### Debranching (NYC samples only)

Debranching has been reported to improve library quality [35]. All samples from the MDA reaction including the positive control were debranched as follows. Residual hexamers in the sample were removed with approximately 30 units of RecJ_f_ (New England Biolabs, Beverly MA). Incubation was for one hour at 37°C followed by phenol:chloroform:isoamyl alcohol extraction and ethanol precipitation. The samples were then incubated in a 50 µl reaction as follows: 25 µl 2X G-Buffer 4 µl Illustra Genomiphi enzyme cocktail, 0.5 mM dNTP's and 4 mM DTT for two hours at to 30°C. Forty units of Bst DNA polymerase large fragment was added to the reaction and incubation proceeded for an additional 2 hours at 65°C. Samples were extracted with phenol:chloroform:isoamyl alcohol and ethanol precipitated. To remove any remaining branched structures, the samples were incubated in a reaction containing 1X NEB buffer 2 and 10 units of T7 endonuclease I (New England Biolabs, Beverly MA) for 30 minutes at 37°C. The samples were phenol:chloroform:isoamyl alcohol (25∶24∶1 v/v) extracted and ethanol precipitated overnight at room temperature.

### Linker Adapted Amplification of Samples (SD samples only)

DNA was sheared using the Covaris S series (Woburn, MA) to 500–800 bp and subsequently blunt-end repaired using BAL-31 nuclease (New England Biolabs, Beverly MA) and T4 DNA Polymerase (New England Biolabs, Beverly MA). I-ceu1 adapters[36] with 5′-phosphate groups were then appended using T4 ligase (New England Biolabs, Beverly MA) followed by amplification using Phusion DNA polymerase (Finnzymes, Espoo Finland). Excess adapters were removed from each sample using gel excision (1% LMP agarose) purification. The amplified DNA fragments were excised from the agarose and recovered with beta-agarase (New England Biolabs, MA), phenol extraction and isopropanol precipitation. The positive control consisted of purified E. coli DNA.

### Gel Purification of DNA

Pooled DNA was gel purified on a 1% LMP gel post stained with SYBR gold. For each sample, an area of the gel corresponding to greater than 500 bp–1000 bp was excised and used for library construction. DNA was recovered from the gel slices with beta-agarase (New England Biolabs, Beverly MA), phenol extraction and isopropanol precipitation.

### Shotgun Library Construction

For NYC samples, two methods were used to produce WGS libraries from the phi29 amplified DNA isolated from the indoor and outdoor filters. In both, DNA was sheared, size selected, and the ends repaired. In the first method, A/B adaptors (Roche technical bulletin 004-2009) were added in an overnight 4°C ligation reaction. Excess adaptors were removed and the adaptorized fragments were recovered as described above. Biotinylated B adaptor for 454 FLX sequencing was incorporated using Phusion DNA polymerase and 15 cycles of PCR. Three PCR reactions were set up for each sample and the reactions consisted of ∼50 ng of adaptorized DNA, 1X Phusion Buffer0.2 mM dNTP's, 50 µm 454 FLX A, 50 µm 454 FLX BioB and 5 units of Phusion DNA polymerase. The biotinylated fragments were recovered from the PCR reaction using streptavidin coated M-280 Dynabeads (Invitrogen, Grand Island, NY) following the manufacturer's supplied protocol. In the second method, libraries were constructed using the 454 rapid library construction kit according to the manufacturer's protocols. For SD samples, the linker adapted DNA libraries were constructed using the 454 rapid library construction kit according to the manufacturer's protocols.

### 16S PCR Amplification (NYC samples only)

Prior to 16S ribosomal PCR, the MDA products were debranched. This step proved necessary to achieve reliable PCR amplification. Post-MDA 16S PCR products were generated using the V3–V5 variable region of the 16S gene. The V3_357F PCR primer sequence was 5′ CCTACGGGAGGCAGCAG and the V5_926R primer sequence was 5′ CCGTCAATTCMTTTRAGT. Three reactions for each sample, including the positive control described in the phi29 amplification section and a no template control, were performed using Phusion DNA polymerase (New England Biolabs, Beverly MA) as described above. The reactions for each sample were pooled, phenol:chloroform:isoamyl alcohol (25∶24∶1 v/v) extracted, ethanol precipitated and sequenced using the 454 Titanium platform.

### Initial processing of metagenomic data

Raw 454 reads were processed to identify and remove artificial replicate sequences [37]. Then, low complexity regions in the remaining reads were identified using DUST [38] and a read was excluded from further analysis if >50% of the length of the read was identified as low complexity. RepeatMasker [39] was used to identify known eukaryotic repeat families (using the RepBase library) on the remaining reads. A read was excluded from further analysis if >50% of the length of the read contained a match to a repeat family.

### Identification of chimeric metagenomic reads

The goal was to identify sequences containing one of the two chimeric rearrangement patterns [40] - inverted sequences and transposed direct sequences. As part of this, we first flagged any sequence that contained an exact ≥25 bp repeat within it (in either the forward or reverse direction) and any sequence with >90% match (over >95% of its length) to this sequence, as chimeras (containing repeats). We chose to be conservative in the flagging process and acknowledge that this constraint may be too strict and can eliminate sequences that contain true repeats (as may be the case in eukaryotes). An all-vs-all BLASTN [41] of the sequences in the sample was then carried out to identify match regions between pairs of sequences. Sequence pairs that had match regions corresponding to the two types of chimeric rearrangements (inverted sequences and transposed direct sequences) were noted as a chimeric pattern. Subsequently, any sequence that had more sequences similar to it with chimeric patterns than those without chimeric patterns, was also flagged as a chimera. This criterion invokes the assumption that chimeric sequences account for only a small fraction of the total reads.

### Taxonomic assignment of metagenomic reads

We used a combination of nucleotide and amino acid searches to assign taxonomy to the metagenomic reads. The nucleotide level searches allowed classification of non-protein coding sequences and were particularly important for identifying human, mouse, and other large eukaryotic components of the data set, while the protein level searches allowed greater sensitivity to detect homology. Thus, first, reads were searched against NCBI non-redundant nucleotide database [42] using BLASTN. A maximum of ten blast hits with an e-value < 1e-5 were retained for each read. If all of the matching database sequences were from eukaryotic genomes, the read was classified as eukaryotic in origin. Then, for all other reads, peptides were predicted and these predictions were annotated (see below). Taxonomic classifications for the peptides (using BLASTP [41]) were obtained as part of the annotation process (including assignments possibly to eukaryotes). The peptide taxonomies were transferred onto the reads: for a read that had multiple peptides with the same taxonomy, that taxonomic classification was transferred onto the read; when the taxonomies conflicted, then the read was assigned a “Mixed” status; if a read had multiple peptides with consistent taxonomy along with unclassified peptides, then the read was assigned that taxonomy. Together with the results from the initial BLASTN step, this process assigned the metagenomic reads to following categories: Archaea, Bacteria, Eukaryota, Virus, Other (synthetic sequences), Mixed, and Unclassified.

### Functional annotation of metagenomic reads

Peptides were predicted on reads using a method that was designed to deal with pyrosequencing errors that result in frame shifts [43]. These peptides were annotated using the JCVI metagenomic annotation pipeline [44] modified to use the Uniref100 database [45] as the subject database for the BLASTP searches.

The bacterial peptides were also searched against KEGG Orthologs (KO) [46], COG [47], Antibiotic Resistance Genes database (ARDB) [48] and Virulence Factor database (VFDB) [49]. Abundance of each KO in a sample was calculated from raw counts and after accounting for differences in read lengths (between samples) and in gene lengths [50]. Individual KO abundances were summed to generate abundances for KEGG functional categories in each sample. COGs were processed in a similar fashion. For ARDB and VFDB searches, only matches with ≥90% identity over ≥90% of the peptide length were considered.

### Mixture modeling

Let *U*, *E*, and *PV* denote the %(G+C) distributions of the reads in the Unclassified, Eukaryotic, and Prokaryotic/Viral groups respectively. Define a new distribution *X* such that X = p*E + (1−p)*PV. We computed an optimal value of *p* for which the symmetric Kullback-Leibler distance [51] between *X* and *U* is minimized.

### Assembly of metagenomic reads

Metagenomic assemblies were computed using Newbler GS De Novo Assembler, version 2.3 (Roche, 454).

### NYC 16S data analysis

Sequences were processed using in-house scripts that deconvoluted the barcoded samples, trimmed barcodes and primers, and removed low quality sequences. Chimeras were detected using Chimeraslayer [52] and removed. Remaining sequences were assigned taxonomy using Ribosomal Database Project classifier [53]. Sub-genus diversity was analyzed using mothur [54]. For this, sequences were aligned using the silva template and the alignment was subsequently trimmed and short sequences removed to produce a master alignment. Since the Operational Taxonomic Unit (OTU) analysis on the full dataset from this master alignment was computationally prohibitively expensive, we used subsampling to calculate rarefaction curves and diversity measures. We generated 25 random samples where each random sample consisted of 10,000 indoor sequences and 10,000 outdoor sequences. These sequences were preclustered and a distance-matrix was generated and subsequently used to produce OTUs at 97% identity (using average linkage clustering).

## Results

### Collection and DNA Isolation

The NYC samples were collected from a modern high rise office building in Midtown Manhattan, while the SD samples were collected from a hospital medical center, a single family ranch-style home with no pets, and a marine pier (Table S1). The NYC collection effort sampled 5.9×10^6^ liters of outdoor air using two modified SpinCon wet cyclone samplers on an outside covered walkway at the 22^nd^ floor air intake plenum (101 m above street level) of the building. In addition, 10.8×10^6^ liters of indoor air was sampled using three modified SpinCon samplers in the mixing room of the same building where filtered outdoor air was mixed with returning indoor air prior to being circulated through the building. As part of the SD sampling, indoor and outdoor air of the hospital was sampled using five SpinCon devices deployed in an HVAC mixing room: three of the devices sampled the recirculating indoor air, while the other two devices sampled the incoming fresh air from the outdoors; 7.2×10^6^ and 3.1×10^6^ liters of indoor and outdoor air were sampled at this site. Indoor air of the house was sampled by running three air samplers placed in the main living area; 0.9×10^6^ liters of air were sampled at this location. The pier sample was collected using four collection devices deployed at the end of the pier, sampling 5.4×10^6^ liters of marine air.

Extensive precautions were taken to reduce the possibility of sample contamination or bacterial growth during sampling. These included a daily decontamination of the samplers between sample batches, inclusion of bacteriostatic antibiotics in the sampling buffer, and UV treatment of all reagents used for DNA isolation and manipulation. Following collection into liquid, samples were filtered onto 3.0 µm and 0.1 µm filters using serial filtration (Figure S1).

Despite the large volumes of air that were sampled, DNA yields were less than 15 ng for each sample, which was inadequate for standard 454 library preparation methods. Therefore it was necessary to perform initial amplification to produce sufficient quantity of DNA for library construction. Amplification requires DNA that is relatively free from non-biological particulates; contamination with foreign DNA either from the sampling or the DNA extraction and isolation should be avoided. To ensure that foreign DNA was not introduced during DNA isolation or amplification, strict protocols were followed, with extensive assessment of potential contamination at each stage. Two methods of amplification were chosen: the NYC samples were amplified by Multiple Displacement Amplification (MDA) using phi29 DNA polymerase [55] and the SD samples were amplified by linker amplification [36]. The amplification products for these samples were quantified using spectroscopy and varied from 100 ng to 1000 ng (Table 1). For these samples, in general, the DNA yields from the 3.0 µm filter could be expected to be at least as high as the yields from the 0.1 µm filter. This is due to a comparatively higher amount of starting material expected on this filter resulting from the capture of large cells (eukaryotic and prokaryotic) in combination with incomplete disaggregation during fractionation, due to free DNA clumping together or with abiotic particles. Table 1 shows this to be the trend for all samples except the indoor hospital sample (likely attributable as a stochastic event).

**Table 1:** DNA yields after amplification.

Sample	Estimated final DNA yield (ng)
NY_INDOOR (0.1 µm)	100
NY_INDOOR (3.0 µm)	200
NY_OUTDOOR (0.1 µm)	500
NY_OUTDOOR (3.0 µm)	1000
SD_IHOSP (0.1 µm)	468
SD_IHOSP (3.0 µm)	259
SD_OHOSP (0.1 µm)	235
SD_OHOSP (3.0 µm)	327
SD_IHOUS (0.1 µm)	110
SD_IHOUS (3.0 µm)	308
SD_SCRPP (0.1 µm)	294
SD_SCRPP (3.0 µm)	370

The MDA products for the NYC samples were quantified by UV spectroscopy and the linker amplification products for the SD samples were quantified by fluorescence spectroscopy.

### Sequence data

Libraries were constructed from the corresponding 3.0 µm and 0.1 µm filters, and these libraries were sequenced using 454 pyrosequencing platform [56]. Reads from the two filters for each location were pooled, generating six metagenomic datasets: NYC indoor (NY_INDOOR), NYC outdoor (NY_OUTDOOR), SD indoor hospital (SD_IHOSP), SD outdoor hospital (SD_OHOSP), SD indoor house (SD_IHOUS), and SD pier (SD_SCRPP). In addition, we were also able to generate 16S rRNA sequence tags from the NYC samples after PCR amplification of the MDA products. Sequence data from all samples have been deposited in the sequence read archive at NCBI [57] and are available under BioProject accession PRJNA218551.

### Metagenomic data

The WGS reads were processed to generate data suitable for downstream analysis. As part of this, three categories of reads were identified and removed: artificial replicates generated by pyrosequencing, reads of low-complexity, and reads containing repeats. An examination of the resulting reads from the MDA samples (NY_INDOOR and NY_OUTDOOR) revealed the presence of chimeric reads. Amplification using phi29 DNA polymerase has been shown to generate chimeric sequences when applied to whole genome amplification from low input DNA amounts; a model based on branch migration can be used to explain the mechanism of chimera formation [40]. Thus, a method using sequence similarity was implemented to detect and remove chimeric sequences from these data. This method was applied to each of the six datasets. At the end of these processing steps, a total of 5,342,939 reads remained (Table S2), and only these were used in subsequent analysis.

### Taxonomy based on metagenomic data

**Figure 1:**
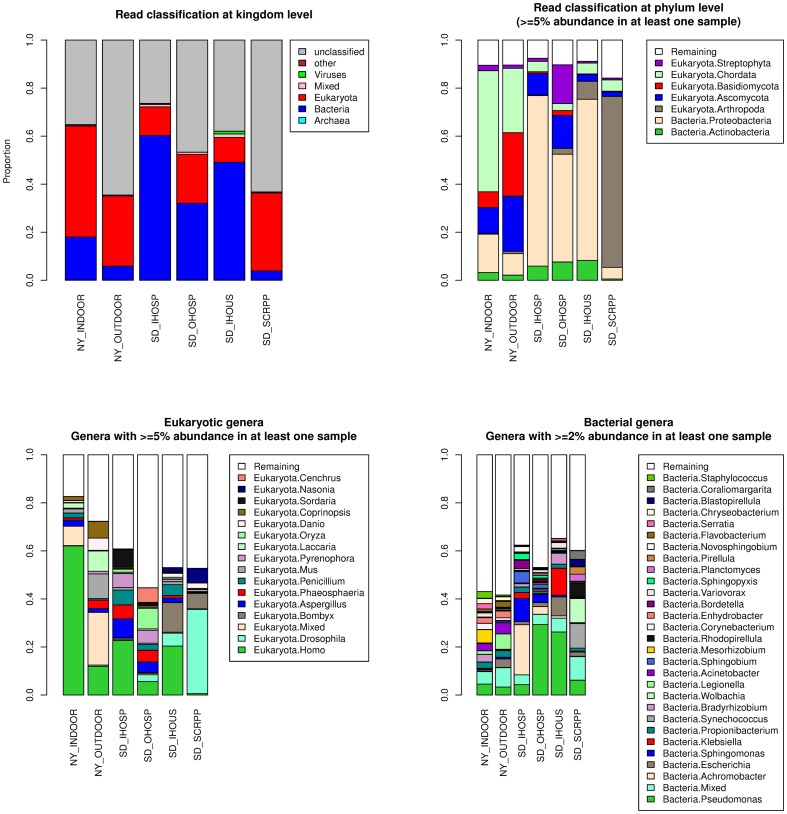
Taxonomic classification of metagenomic reads.

A BLAST based method was used to assign taxonomy to the reads. On average, 54% of the reads in a sample could be assigned taxonomy (Table 2, Figure 1, Table S3), with SD_IHOSP having the highest (74%) and NY_OUTDOOR having the lowest (35%) assignment proportions. Sequences from eukaryota and bacteria dominate the portion of data that can be assigned taxonomy. Eukaryotic sequences make up 71%, 82%, and 88% respectively of assigned sequences in NY_INDOOR, NY_OUTDOOR, and SD_SCRPP, with the remaining assigned sequences in these samples primarily from bacteria. On the other hand, SD_IHOSP, SD_OHOSP, and SD_IHOUS are dominated by bacteria (81%, 60%, and 79% respectively), with the remaining assigned sequences in these samples almost exclusively from eukaryota. For the eukaryotic portion of these samples, human and fungal (*Ascomycota* and *Basidiomycota*) sequences are predominant in indoor samples, while outdoor samples contain these together with a more diverse mix of rodent (mouse), plant (*Streptophyta*), fish, and insect (*Arthropoda*) sequences.

**Table 2:** Taxonomic classification of metagenomic reads.

	NY_INDOOR	NY_OUTDOOR	SD_IHOSP	SD_OHOSP	SD_IHOUS	SD_SCRPP
Archaea	1,048	269	286	606	90	324
Bacteria	256,691	55,681	347,562	263,396	192,326	44,092
Eukaryota	663,225	281,601	68,998	167,883	40,334	375,198
Mixed	6,012	1,915	5,975	7,010	5,936	2,356
Viruses	958	495	1,926	514	4,186	2,026
Other	1,084	1,389	491	167	206	67
Unclassified	504,660	620,628	152,468	384,538	148,683	729,639
Total	1,433,678	961,978	577,706	824,114	391,761	1,153,702

*Mixed* refers to a read that had matches to multiple kingdoms and could not be definitively assigned to one kingdom. *Other* refers to sequences that could not be identified (NCBI taxonomy ID 32644), mostly synthetic constructs. *Unclassified* refers to a read that could not be assigned to any kingdom (i.e. had no match in the reference databases). Kingdom taxonomy:

*Proteobacteria* is the dominant bacterial phylum in all samples, ranging from 46% of bacterial sequences (in SD_SCRPP) to 87% of bacterial sequences (in SD_IHOSP). *Actinobacteria* and *Bacteroidetes* phyla are also well represented in most of these samples. SD_SCRPP has a high representation (>10% of bacterial sequences) from two marine associated bacterial phyla (*Cyanobacteria* and *Planctomycetes*). At the genus level, however, the bacterial genera representation and abundance vary between samples (Table S3). The top three represented genera in the samples (as a proportion of all bacterial sequences in the sample) are: NY_INDOOR – *Mesorhizobium* (5.5%), *Pseudomonas* (4.5%), and *Bradyrhizobium* (3.1%); NY_OUTDOOR – *Legionella* (6.4%), *Acinetobacter* (4.6%), and *Escherichia* (3.5%); SD_IHOSP – *Achromobacter* (20.9%), *Sphingomonas* (9.5%), and *Sphingobium* (4.7%); SD_OHOSP – *Pseudomonas* (29.3%), *Sphingomonas* (3.3%), and *Achromobacter* (3.2%); SD_IHOUS – *Pseudomonas* (26.2%), *Klebsiella* (11.2%), and *Escherichia* (7.9%); SD_SCRPP – *Synechococcus* (10.3%), *Wolbachia* (10%), and *Pseudomonas* (6.1%). The bacterial populations represented by these samples have a long tail of low abundant genera. This measure of diversity is supported by the observation that bacterial genera that have <2% abundance (in the corresponding sample) account for 64%, 66%, 43%, 60%, 42%, and 46% respectively of the bacterial sequences in NY_INDOOR, NY_OUTDOOR, SD_IHOSP, SD_OHOSP, SD_IHOUS, and SD_SCRPP.

A substantial number of metagenomic reads in these samples (45% on average) have no taxonomic assignments (Table 2). To explore the likely taxonomic origin of these unclassified reads, we compared GC composition of reads in the unclassified group (*U*) to that of reads in the eukaryotic group (*E*) and the prokaryotic/viral group (*PV*) (Figure S2). Assuming that reads from the unclassified group actually belong to one of *E* or *PV* groups, the *U* distribution can be interpreted as a mixture distribution obtained by sampling from the *E* and *PV* distributions. We computed the probability *p* of sampling from the *E* distribution (and thus probability 1−*p* of sampling from the *PV* distribution) so as to minimize the (symmetric) Kullback-Leibler distance[51] between the resulting mixture distribution and the *U* distribution. These calculations suggest that a vast majority of the unclassified reads in NY_OUTDOOR (*p = 0.93*) and SD_SCRPP (*p = 0.89*), and to a lesser extent unclassified reads in SD_OHOSP (*p = 0.78*) and NY_INDOOR (*p = 0.75*), may be eukaryotic in origin (Figure S3).

The six datasets were assembled individually to assess recovery of long genomic sequences from these metagenomes. However, the assemblies were fragmented, with average contig sizes less than 1 kbp for each sample (Table S4). At 96% identity threshold, SD_IHOSP had the largest proportion of assembled reads (65.2%) while NY_INDOOR had the lowest proportion (7.1%). The low degree of these assemblies attest to the diverse nature of the metagenomes being sampled and combined with an insufficient depth of sequencing of the associated microbial communities.

### 16S rRNA based taxonomic profiling of NYC samples

**Figure 2:**
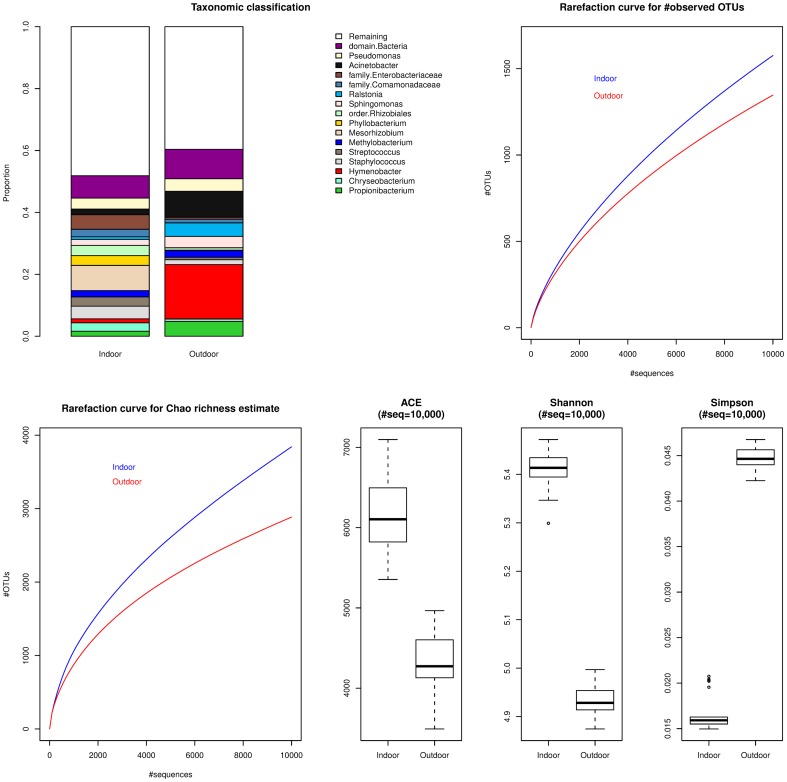
Taxonomic classification and diversity of the NYC 16S data. In the stacked barcharts, only those taxonomic groups that have ≥2% abundance in at least one of the samples is reported. The rarefaction curves along with the richness and diversity estimates were calculated using mothur and were based on averages of 25 random samples of 10,000 sequences from each dataset.

410,373 and 230,506 sequences respectively were generated from NY_INDOOR and NY_OUTDOOR samples. A small fraction of these sequences were identified as chimeras: 12,503 (3.0%) and 5,287 (2.2%) respectively in NY_INDOOR and NY_OUTDOOR, and these were removed. Chloroplast sequences comprised approximately 6% of the 16S sequences in NY_INDOOR and 8% of the sequences from NY_OUTDOOR, and these were also removed prior to further analysis. At the phylum level, NY_INDOOR is dominated by *Proteobacteria* (60%) and *Firmicutes* (14%), while NY_OUTDOOR is dominated by *Proteobacteria* (52%) and *Bacteroidetes* (22%). *Actinobacteria* are nearly equally abundant in both samples comprising 5% of NY_INDOOR and 7% of NY_OUTDOOR. *Mesorhizobium* (8.2%), *Staphylococcus* (4.0%), and *Pseudomonas* (3.0%) are the most abundant genera in NY_INDOOR, while *Hymenobacter* (17.6%), *Acinetobacter* (8.4%), and *Propionibacterium* (4.8%) are the most abundant genera in NY_OUTDOOR (Figure 2). Overall, 48% of NY_INDOOR sequences and 40% of NY_OUTDOOR sequences were assigned to genera that have <2% abundance in both samples. NY_INDOOR has a higher richness and diversity compared to NY_OUTDOOR (Figure 2).

For these two samples, the 16S based abundances of some taxonomic groups are different from their abundances inferred from metagenomic data. This can be primarily attributed to a lack of sequenced genomes that can serve as adequate near-neighbour references, but for which 16S sequences are available. These groups include *Hymenobacter (Bacteriodetes), Paracoccus (Alphaproteobacteria)*, *Enhydrobacter (Alphaproteobacteria)*, and *Phyllobacterium (Alphaproteobacteria)*.

### Functional analysis of metagenomic data

A total of 3,777,158 proteins were predicted from reads in prokaryotic, viral, and unclassified groups, and subsequently annotated. 45% of them could be assigned a function or had a match to a hypothetical protein in the reference database (Table S5). SD_IHOSP had the highest assignment proportion (71%) while NY_OUTDOOR had the lowest assignment proportion (14%). Those samples that had a larger predicted contribution of eukaryotes to the unclassified group, tended to have lower proportions of functional assignments.

**Figure 3:**
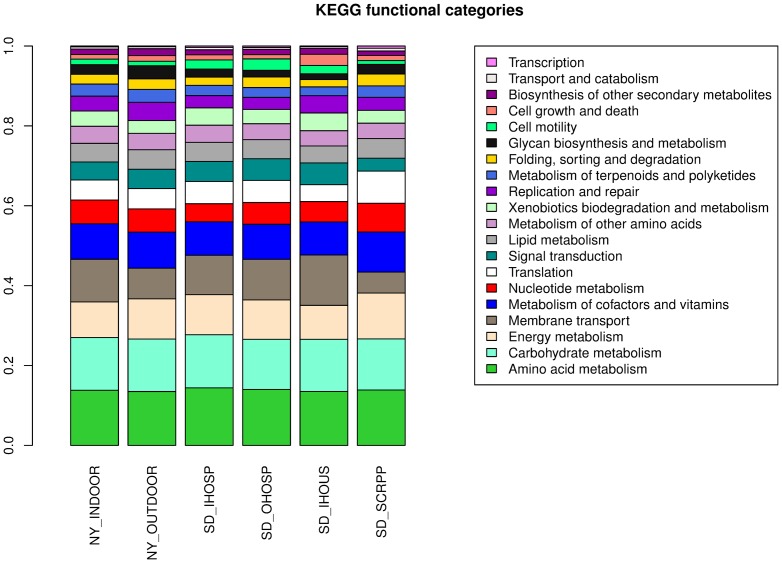
Abundances of KEGG functional categories.

**Figure 4:**
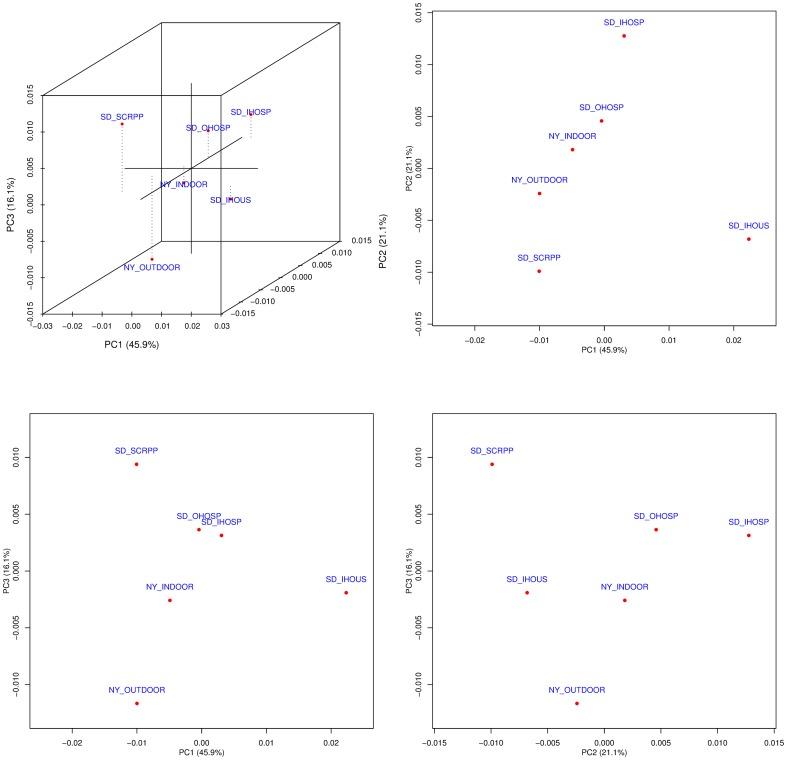
Ordination of the six metagenomic samples by principal component analysis based on KO abundance profiles. The first three principal components (PC1, PC2, and PC3) account for 83.1% of the total variation. The dashed lines in the 3D plot shows the height (PC3) of the sample points when projected on to the PC1-PC2 plane.

In order to assess the functional composition of bacteria in these samples, KEGG Orthology (KO) [46] abundances were calculated using the bacterial proteins (Table S6). The six samples show similar abundance profiles of KEGG categories (obtained by summing KO abundances), with an average correlation of 0.95 between pairwise sample profiles (Figure 3). In general, metabolism and transport (amino acid, carbohydrate and energy conversion) are the top functional categories followed by translation and signal transduction. A higher resolution ordination of the samples was also carried out by a principal component analysis using KO abundance profiles. While the first three principal components PC1, PC2, and PC3 explain 83.1% of the total variation, the ordination does not suggest obvious sample groupings or a separation across indoor and outdoor environments (Figure 4). It is however possible to identify the KOs that contribute the most along each principal component axis and the corresponding outlier samples. Notably, SD_IHOUS is an outlier along PC1 and this is primarily driven by a higher proportion of DNA replication proteins (K02314 and K02315) in this sample. Along PC3, SD_SCRPP has a higher abundance of arylsulfatases (K01130), while NY_OUTDOOR has a higher abundance of beta-lactamase (K01467) and tetracycline resistance proteins (K08151). A similar observation is made by an analysis of COGs [47] (Table S6).

Bacterial proteins were searched for presence of virulence genes and antibiotic resistance genes in these samples (Table S7). There was no evidence of significant matches to true virulence genes [58] directly implicated in pathogenicity. The antibiotic resistance gene searches, however, return matches to beta-lactamases and tetracycline resistance genes in several samples, with NY_OUTDOOR having comparatively higher amounts (consistent with the KEGG based analysis).

## Discussion

Air is a low biomass environment and therefore the application of WGS methods to study airborne microbial communities is a challenge. A previously reported metagenome study of airborne microbes in indoor environments consisted of the extraction and sequencing of DNA collected on air filters that are part of air-handling units in buildings [59]. Here we describe protocols for the direct sampling of air from indoor and outdoor environments. As obtaining enough nucleic acid to conduct sequencing of these samples requires long sampling times, suitable collection protocols are necessary to avoid growth during sample collection, which would otherwise reflect culture biases rather than the source environment. Abiotic particulates present in the samples contribute to the challenge of isolating DNA that is not refractory to amplification. Despite these limitations we were able to develop sampling and amplification strategies to enable the study of this low biomass environment. We presented two different amplication strategies, phi29 MDA and linker amplification, to generate enough material for pyrosequencing to assess the genomic content of airborne metagenomes. Transposon based methods, such as Nextera, for simultaneously fragmenting and tagging DNA recently became available. These methods have been used to sequence phage and viral genome samples containing <1 ng of DNA [60], [61]. The utility of this method for sequencing low biomass metagenomic samples is promising, however, this type of method may miss genomes present in the sample which contain few or no recognition sites for the transposon resulting in DNA that is not fragmented or poorly fragmented, or contain multiple recognition sites for the transposon causing over-fragmentation of the DNA. The methods presented here avoid this issue.

Analysis of sequence data generated from these samples reveals a high degree of diversity of the sampled microbes. This is supported by their taxonomic profiles (both metagenomic and 16S profiles contain long tails of many low-abundant organisms), a high number of unclassified sequences and hypothetical proteins, and the highly fragmented nature of nucleotide assemblies. Furthermore, sequenced bacterial genomes do not serve as adequate near-taxonomic neighbor references for these samples, as evidenced by a low-number of bacterial reads with high identity nucleotide matches to these genomes (Figure S4, Table S8).

While the six samples have different genera abundance profiles, they share common features as well. These data include human associated bacteria in many of the samples. For instance, *Propionibacterium*, a group associated with human skin microbiome, is found in all indoor samples. In addition, the hospital samples had notable numbers of human associated medically relevant organisms, several of which are known hospital associated organisms, including *Klebsiella* and *Bordetella*. On the other hand, many of the abundant bacterial genera from the SD_SCRPP sample were of marine origin (including *Synechococcus*, *Plantomyces*, *Pirellula*, and *Rhodopirellula*), showing that the environment influences the types of microbes found in the overlying air.

Human, fungal, insect, and rodent sequences constitute the eukaryotic portion of these data with human and fungal sequences present in relatively larger amounts in the indoor samples. The SD_SCRPP sample had a sizeable number of sequences from *Wolbachia*, an endosymbiont of insects including *Drosophila*, which constitutes a large portion of the eukaryotic sequences in SD_SCRPP. There are several potential sources of eukaroytic DNA including cells, sloughed tissue (such as skin), and DNA adhered to particulates that were captured on the filters.

The data presented here support the notion that sources of airborne bacteria in indoor and outdoor environments include water, soil, vegetation, and fauna [1], [16], [25]. However, at the functional level, no obvious grouping by environments (indoor and outdoor) or sampling locations is evident for these samples, and sample separation is driven by taxonomic composition. For instance, the higher abundance of arylsulfatases in the SD_SCRPP sample is due to their occurrence in the corresponding marine organisms.

The framework presented here, in combination with further experiments including repeated sampling and gene expression data generation, can be used to identify airborne bacteria that are metabolically active, and to differentiate between transient members and those that use air as a habitat.

## Supporting Information

Figure S1 **Sampling of airborne microorganisms.**
(PDF)

Figure S2 **GC composition profile for the unclassified group (U), eukaryotic group (E), and prokaryotic/viral group (PV).**
(PDF)

Figure S3 **Best fit curves for the mixture modeling using the optimal value of** ***p*** **.**
(PDF)

Figure S4 **Proportion of prokayotic and viral reads recruited to sequenced genomes.**
(PDF)

Table S1 **Metadata associated with sample collection locations.**
(PDF)

Table S2 **Quality filtering of metagenomic sequence data.**
(PDF)

Table S3 **Taxonomic composition (at phylum and genus levels) of samples.**
(XLSX)

Table S4 **Newbler assembly statistics for the samples.** Individual assemblies at two different minimum nucleotide identity thresholds (96% and 86%) were computed.
(PDF)

Table S5 **Protein predictions on reads from prokaryotic, viral, and unclassified groups.**
(PDF)

Table S6 **Abundances of KEGG and COG protein groups in the samples.**
(XLSX)

Table S7 **Search results against Virulence Factor Database (VFDB) and Antibiotic Resistance Genes Database (ARDB).**
(XLSX)

Table S8 **Mapping of metagenomic reads to sequenced genomes.** A read was considered as mapping to a sequenced genome if its BLASTN match had > = 80% identity over > = 90% of the read length.
(XLSX)
