# Supplementary figures and images for: A Metagenomic Framework for the Study of Airborne Microbial Communities
Source: PLoS One. 2013 Dec 11;8(12):e81862. doi: 10.1371/journal.pone.0081862 (PMC3859506; doi:10.1371/journal.pone.0081862)

Sampling Buffer: ~10 ml antimicrobial cocktail

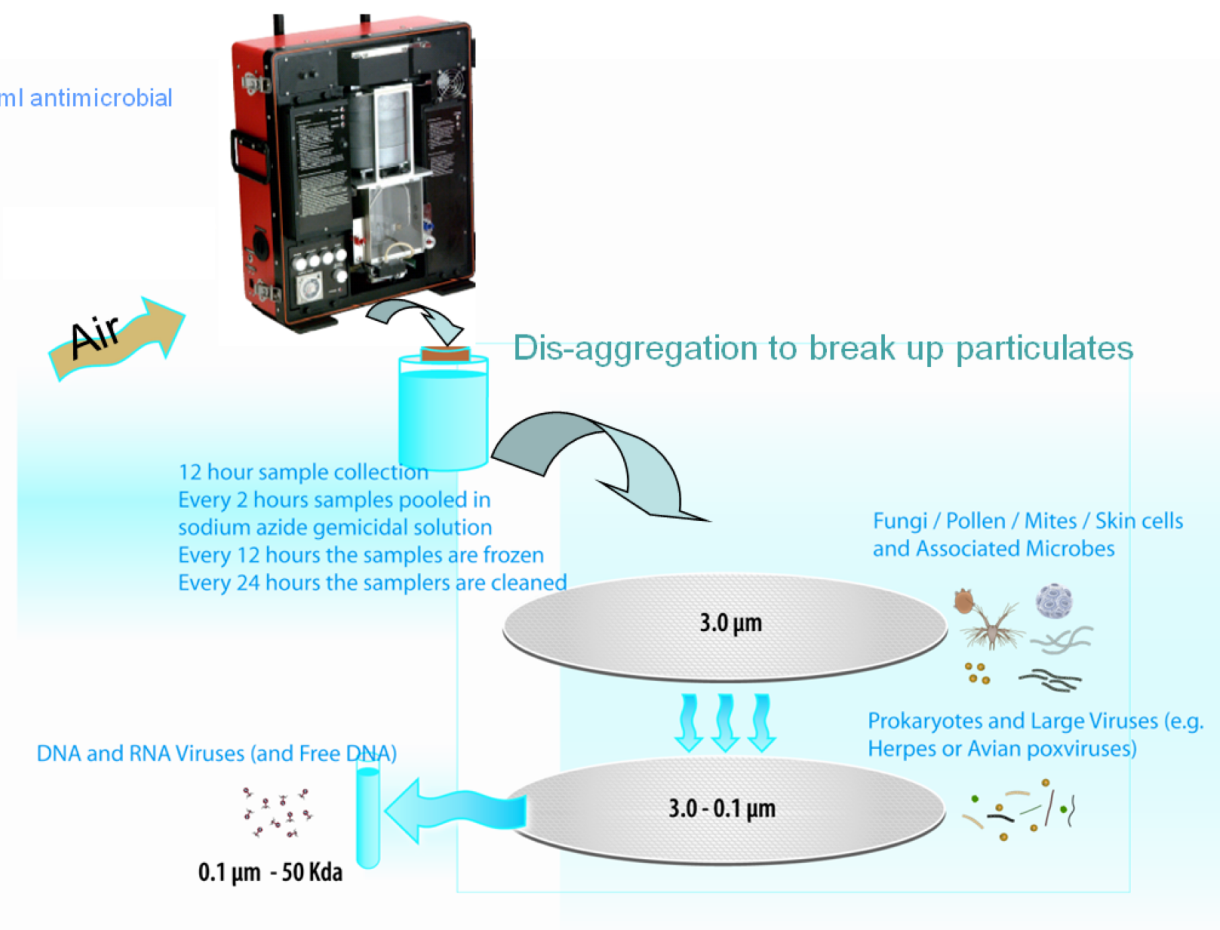

**Figure S1: Sampling of airborne microorganisms.**

Supplement: Figure S1 — Sampling of airborne microorganisms. (PDF) [file pone.0081862.s001.pdf]

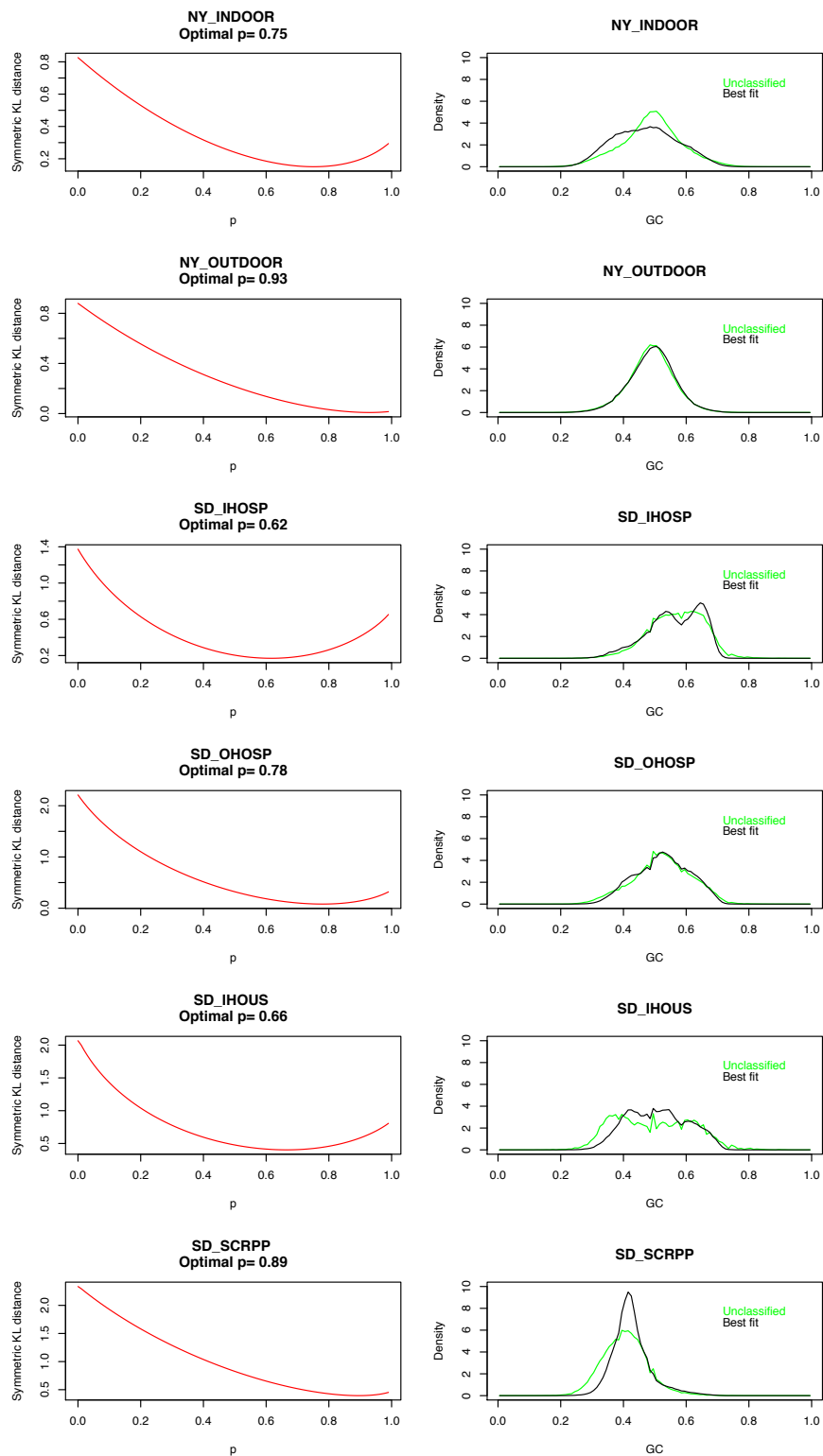

**Figure S3: Best fit curves for the mixture modeling using the optimal value of  $p$ .**

Supplement: Figure S3 — Best fit curves for the mixture modeling using the optimal value of p . (PDF) [file pone.0081862.s003.pdf]

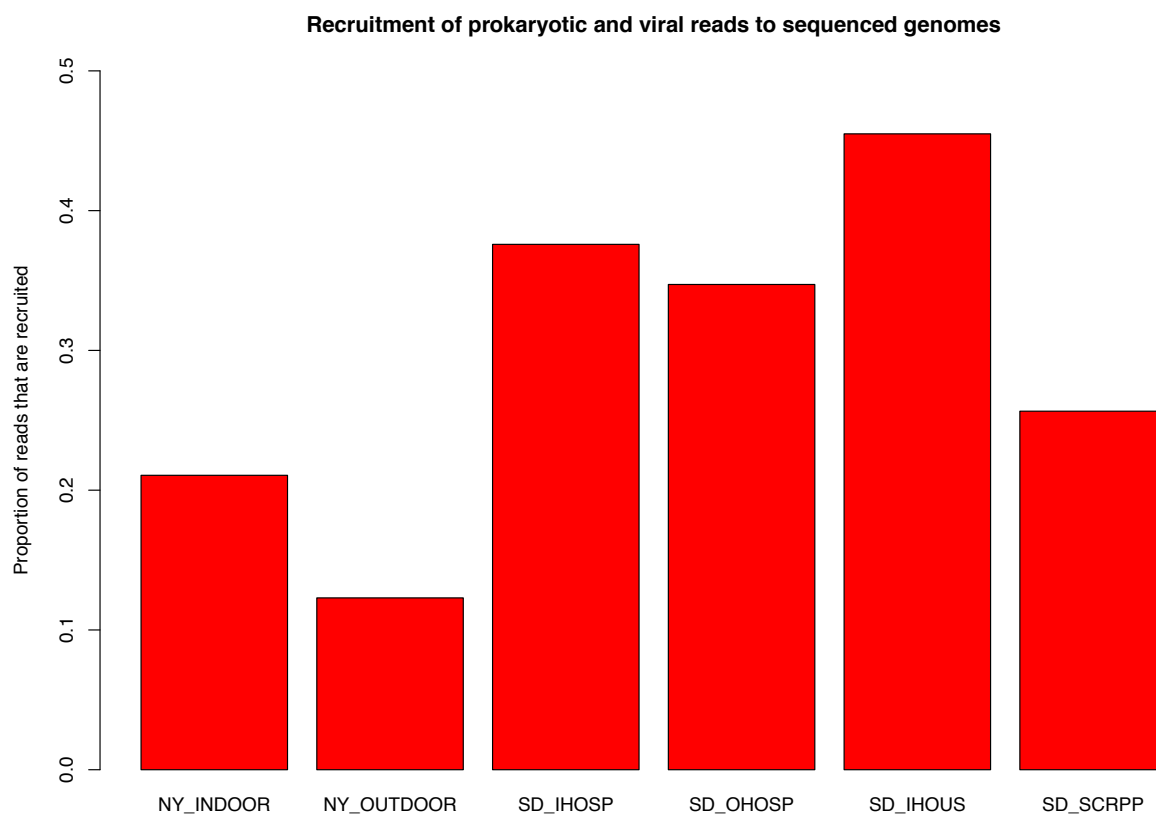

**Figure S4: Proportion of prokaryotic and viral reads recruited to sequenced genomes.**

Supplement: Figure S4 — Proportion of prokayotic and viral reads recruited to sequenced genomes. (PDF) [file pone.0081862.s004.pdf]
