# Supplementary material for: A Metagenomic Framework for the Study of Airborne Microbial Communities
Source: PLoS One. 2013 Dec 11;8(12):e81862. doi: 10.1371/journal.pone.0081862 (PMC3859506; doi:10.1371/journal.pone.0081862)
Supplement: Figure S2 — GC composition profile for the unclassified group (U), eukaryotic group (E), and prokaryotic/viral group (PV). (PDF) [file pone.0081862.s002.pdf]

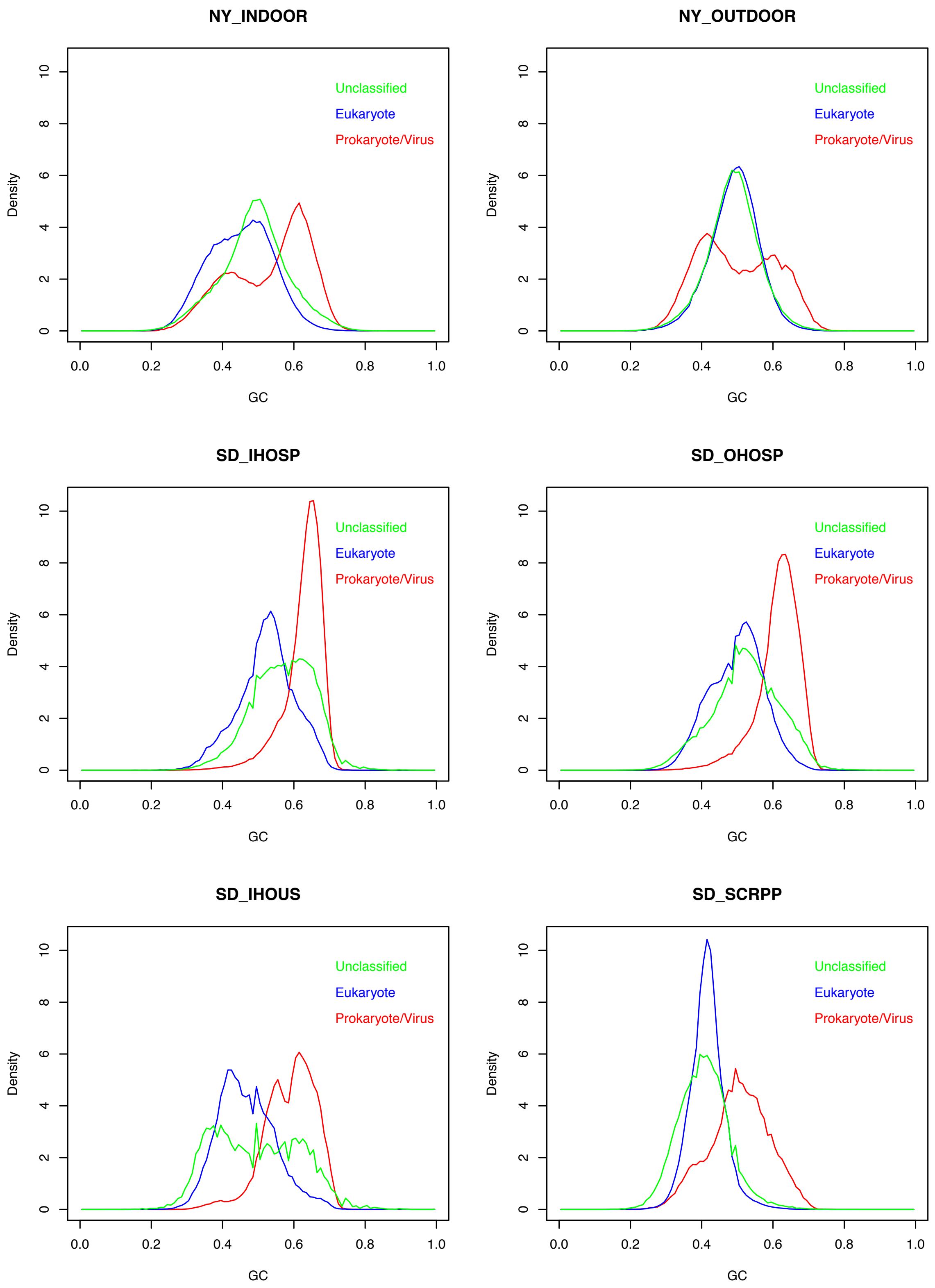

Figure S2: GC composition profile for the unclassified group (U), eukaryotic group (E), and prokaryotic/viral group (PV).
