# Supplementary material for: A Metagenomic Framework for the Study of Airborne Microbial Communities
Source: PLoS One. 2013 Dec 11;8(12):e81862. doi: 10.1371/journal.pone.0081862 (PMC3859506; doi:10.1371/journal.pone.0081862)
Supplement: Table S1 — Metadata associated with sample collection locations. (PDF) [file pone.0081862.s005.pdf]

Table S1. Metadata associated with sample collection locations.

Location	Sampling dates	Sample	Avg. temp.	Avg. Rel. Humidity	Prevail Wind Speed	Prevail. Wind Direction	Comments
NYC Building (near 40.761N, 73.983W)	10 - 16 NOV 2007	NY INDOOR	19.5 °C	40%	N/A	N/A	Light rain on 13 and 15 of November
		NY OUTDOOR	8.3 °C	58%	2.4 m/s	WNW	Light rain on 13 and 15 of November
SD House (near 32.801N, 117.077W)	5 - 7 JUN 2010	SD INDOOR HOUSE	22 °C	53%	N/A	N/A	Clear, no rain
SD Medical Center (near 32.753N, 117.163W)	8 - 21 JUN 2010	SD INDOOR HOSPITAL	21.5 °C	44%	N/A	N/A	Morning cloud cover, no rain
		SD OUTDOOR HOSPITAL	18.5 °C	74%	3.2 m/s	W	Morning cloud cover, no rain
SD Pier (at 32.867N, 117.257W)	5 - 10 JUL 2010	SD PIER	17.5 °C	89%	2.8 m/s	WNW	Low cloud cover all five days, no rain
