# Supplementary material for: A Metagenomic Framework for the Study of Airborne Microbial Communities
Source: PLoS One. 2013 Dec 11;8(12):e81862. doi: 10.1371/journal.pone.0081862 (PMC3859506; doi:10.1371/journal.pone.0081862)
Supplement: Table S2 — Quality filtering of metagenomic sequence data. (PDF) [file pone.0081862.s006.pdf]

Table S2. Quality filtering of metagenomic sequence data.

	NY_INDOOR	NY_OUTDOOR	SD_IHOSP	SD_OHOSP	SD_IHOUS	SD_SCRPP
Initial Number of Reads	3227901	3181781	1495317	1411042	1853228	1583257
Post Artificial Replicate Filtering	2567243	2108534	628593	849437	456286	1264338
Post Low Complexity Filtering and Repeat Masking	1598261	1197407	593912	842254	416728	1256113
Post Chimera Detection	1433678	961978	577706	824114	391761	1153702
