# Supplementary material for: A Metagenomic Framework for the Study of Airborne Microbial Communities
Source: PLoS One. 2013 Dec 11;8(12):e81862. doi: 10.1371/journal.pone.0081862 (PMC3859506; doi:10.1371/journal.pone.0081862)
Supplement: Table S4 — Newbler assembly statistics for the samples. Individual assemblies at two different minimum nucleotide identity thresholds (96% and 86%) were computed. (PDF) [file pone.0081862.s008.pdf]

Table S4. Newbler assembly statistics for the samples. Individual assemblies at two different minimum nucleotide identity thresholds (96% and 86%) were computed.

	NY_INDOOR	NY_OUTDOOR	SD_IHOSP	SD_OHOSP	SD_IHOUS	SD_SCRPP
**Input Reads**	1,433,624	961,778	577,705	824,114	391,761	1,153,701
**Input Bases**	502,582,263	293,707,031	203,995,362	242,186,832	123,622,093	336,367,571
**Assembled Reads**	101,165	134,720	376,915	237,068	195,626	229,096
**Assembled Bases**	24,808,957	28,987,750	139,152,571	78,456,095	70,184,338	69,211,834
**%Assembled Reads**	7.06%	14.01%	65.24%	28.77%	49.94%	19.86%
**%Assembled Bases**	4.94%	9.87%	68.21%	32.39%	56.77%	20.58%
**#contigs**	9119	10573	24,697	21,424	14,383	28,282
**Average Contig size**	854	791	808	646	685	789
**N50 Contig size**	869	765	722	589	642	740
**#contigs >500 bp**	4283	4070	12,884	11,312	7,902	11,130
**Largest Contig size**	5471	3699	42,057	6,830	11,323	8,678

(A) 96% identity threshold

	NY_INDOOR	NY_OUTDOOR	SD_IHOSP	SD_OHOSP	SD_IHOUS	SD_SCRPP
**Input Reads**	1,433,624	961,778	577,705	824,114	391,761	1,153,701
**Input Bases**	502,582,263	293,707,031	203,995,362	242,186,832	123,622,093	336,367,571
**Assembled Reads**	122,561	164,179	379,767	247,730	200,480	242,346
**Assembled Bases**	29,327,426	35,836,449	139,649,082	81,066,905	71,300,383	72,400,290
**%Assembled Reads**	8.55%	17.07%	65.74%	30.06%	51.17%	21.01%
**%Assembled Bases**	5.84%	12.20%	68.46%	33.47%	57.68%	21.52%
**#contigs**	11227	13674	25,062	24,078	15,107	31,701
**Average Contig size**	856	799	808	658	701	793
**N50 Contig size**	874	784	725	601	659	744
**#contigs >500 bp**	5252	5631	13,018	12,359	8,238	12,059
**Largest Contig size**	5645	4058	42,057	6,832	11,323	8,593

(B) 86% identity threshold
