# Supplementary material for: A Metagenomic Framework for the Study of Airborne Microbial Communities
Source: PLoS One. 2013 Dec 11;8(12):e81862. doi: 10.1371/journal.pone.0081862 (PMC3859506; doi:10.1371/journal.pone.0081862)
Supplement: Table S5 — Protein predictions on reads from prokaryotic, viral, and unclassified groups. (PDF) [file pone.0081862.s009.pdf]

Table S5. Protein predictions on reads from prokaryotic, viral, and unclassified groups.

	**#reads**	**#peptides on non-euk reads**	**#peptides that have functional assignment or are conserved hypotheticals**
NY_INDOOR	1433678	760301	298657
NY_OUTDOOR	961978	583993	81774
SD_IHOSP	577706	577849	410781
SD_OHOSP	824114	672975	356118
SD_IHOUS	391761	359701	238543
SD_SCRPP	1153702	822339	317785
